# Cannabidiol Alleviates Imiquimod-Induced Psoriasis by Inhibiting JAK2–STAT3 in a Mouse Model

**DOI:** 10.3390/biomedicines12092084

**Published:** 2024-09-12

**Authors:** Min-Seo Kim, Ji-Hyun Lee, Sae-Woong Kim, Chul-Hwan Bang

**Affiliations:** 1 Department of Medical Sciences, Graduate School of The Catholic University of Korea, Seoul 06591, Republic of Korea; ms5999@catholic.ac.kr (M.-S.K.); ejee@catholic.ac.kr (J.-H.L.); 2 Department of Dermatology, Seoul St. Mary’s Hospital, College of Medicine, The Catholic University of Korea, Seoul 06591, Republic of Korea; 3 Department of Urology, Seoul St. Mary’s Hospital, College of Medicine, The Catholic University of Korea, Seoul 06591, Republic of Korea; ksw1227@catholic.ac.kr; 4 Green Medicine Co., Ltd., Seoul 06591, Republic of Korea

**Keywords:** cannabidiol, CBD, psoriasis, JAK–STAT pathway, JAK2, STAT3

## Abstract

Cannabidiol (CBD), a non-psychoactive compound from *Cannabis sativa*, has shown efficacy in treating psoriasis, a chronic inflammatory skin disease affecting 1–3% of the global population; however, the mechanisms remain unclear. This study investigated CBD’s effects on imiquimod (IMQ)-induced psoriasis in mice, which were divided into five groups: Control, IMQ, Clobetasol, 0.01% CBD, and 0.1% CBD. After inducing psoriasis with IMQ, clobetasol or CBD was applied. Psoriasis severity was assessed using the Psoriasis Area and Severity Index (PASI), with histopathological changes examined via hematoxylin and eosin staining. Gene expression of inflammatory markers (*Il1b*, *Il6*, *Il12b*, *Il17a*, *Il22*, and *Tnf*) was analyzed by RT-PCR, while protein levels of signal transducer and activator of transcription (STAT)3, P-STAT3, Janus kinase (JAK)2, and JAK3 were evaluated through western blot and immunohistochemistry. The results demonstrated that CBD significantly reduced PASI scores, epidermal thickness, keratosis, hyperproliferation, and inflammation. Moreover, CBD inhibited the IL-23 receptor-mediated JAK2–STAT3 signaling pathway, leading to the downregulation of *Il1b*, *Il6*, *Il12b*, *Il17a*, *Il22*, and *Tnf* expression. These findings suggest that CBD effectively alleviates psoriasis-like symptoms in mice and may serve as a promising therapeutic agent for psoriasis by targeting the JAK2–STAT3 pathway.

## 1. Introduction

Cannabidiol (CBD) is a type of cannabinoid that has received increasing attention among recent studies. Cannabinoids derived from *Cannabis sativa* can be classified into three categories: endocannabinoids, phytocannabinoids, and synthetic cannabinoids. The most well known of these are the plant-derived phytocannabinoids, which include tetrahydrocannabinol (THC) and CBD [1]. THC, which is known to have psychoactive effects, can affect the mental health of users, triggering hallucinations, delusions, and psychosis [2,3]. On the other hand, CBD has the advantage of not having psychoactive properties [4], unlike THC, and it has shown promise for the alleviation of various diseases [5]. In recent years, CBD has garnered attention for its potential in treating various skin conditions, such as psoriasis [6]. Recent studies have suggested that CBD’s effects on skin diseases such as allergic contact dermatitis, acne, and psoriasis are attributed to the inhibition of pro-inflammatory cytokines such as TNF-α, IL-1β, IL-6, and IL-17A [5,7,8]. Moreover, clinical trials for the treatment of psoriasis have demonstrated the effectiveness and safety of CBD [9]. CBD’s therapeutic potential is further supported by its physicochemical properties, particularly its lipophilicity and chemical stability [10]. Its lipophilic nature facilitates penetration into lipid-rich environments such as the skin, making it well-suited for topical formulations. As such, CBD shows promise as a potential psoriasis treatment, but its molecular mechanisms and targets remain unclear.

Psoriasis, a chronic inflammatory skin disease, affects approximately 1–3% of the global population [11,12]. Psoriasis results from overactivity of cytokines such as IL-6, IL-17, and IL-23, which causes keratinocyte hyperproliferation. This leads to the onset of thickened, inflamed plaques with silvery scaling; along with parakeratosis, hyperkeratosis, and migration of inflammatory leukocytes into both the dermis and epidermis [13]. Psoriasis has considerable adverse effects on patient quality of life and is associated with depression and suicidal tendencies in affected individuals [14,15]. Although the exact mechanism of psoriasis’ pathogenesis is not yet fully understood, the IL-17/IL-23 axis is now widely acknowledged to be the main pathological mechanism responsible for psoriasis [16,17]. Additionally, however, the Janus kinase (JAK)–signal transducer and activator of transcription (STAT) signaling pathway has been implicated in signaling of the IL-17/IL-23 axis. Binding of the IL-23 receptor activates JAK2 and tyrosine kinase 2 (TYK2) and phosphorylates STAT3. Phosphorylated STAT3 dimerizes and enters the nucleus, where it triggers the production of IL-17 and IL-22. A number of new drugs are currently under development, with these pathways as the target [18,19].

In this study, we examined the functioning of CBD in the JAK–STAT pathway through the use of an imiquimod (IMQ)-induced psoriasis mouse model and explored its potential therapeutic benefits for individuals with psoriasis.

While previous studies have examined IL-17 or T cell inhibition in psoriasis, there is limited research on the upstream pathways driving these effects. Our study extends beyond cytokine reduction to explore the JAK2–STAT3 pathway, elucidating the specific mechanisms by which CBD exerts its therapeutic effects. This approach offers a deeper understanding of how CBD modulates immune responses, presenting new opportunities for therapeutic intervention [8,20].

## 2. Materials and Methods

### 2.1. Drug Preparation

The CBD cream was prepared by first dissolving CBD in dimethyl sulfoxide (DMSO) and then thoroughly mixing it with white petrolatum. During the determination of the appropriate concentration, initial experiments tested concentrations of 1% and higher. However, these higher concentrations led to increased irritation, prompting the selection of the current lower concentrations. To create the 0.01% and 0.1% CBD creams, specific amounts of CBD were accurately added to the white petrolatum to achieve the desired concentrations, ensuring a consistent therapeutic dose of 62.5 mg per mouse.

### 2.2. Animals and Treatment

All animal research procedures were reviewed and approved by the Institutional Animal Care and Use Committee of the School of Medicine and the Animal Research Ethics Committee of The Catholic University of Korea. The procedures complied with the Laboratory Animals Welfare Act and were conducted in accordance with the Guide for the Care and Use of Laboratory Animals (approval no. CUMC-2023-0006-03).

**Figure 1:**
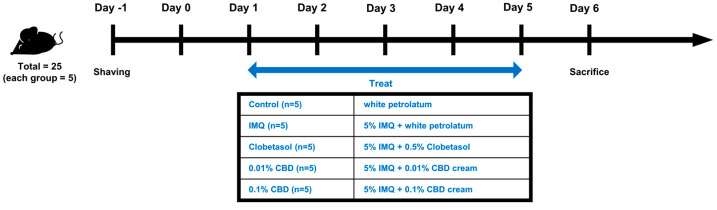
Experimental design and schedule. Five groups were established for this study: Control, IMQ, Clobetasol, 0.01% CBD, and 0.1% CBD; each consisting of five mice. The dorsal skin of all mice was shaved and, two days later, the Control group received white petrolatum for five consecutive days. The other groups were treated with 5% IMQ cream for five consecutive days, followed by their respective treatments four hours later: 62.5 mg of white petrolatum for the IMQ group, 120 mg of 0.5% clobetasol cream for the Clobetasol group, and 62.5 mg of either 0.01% or 0.1% CBD cream for the 0.01% and 0.1% CBD groups, respectively. IMQ, imiquimod.

In our study, we used 5-week-old male C57BL/6 mice (SLC, Inc., Shizuoka, Japan). The total number of mice used was 25, with 5 mice per group. In the Control group, a topical daily dose of 62.5 mg of white petrolatum (Vaseline^®^, Unilever, London, UK) was applied to the shaved dorsal area for 5 consecutive days. Meanwhile, in the IMQ, Clobetasol, 0.01% CBD, and 0.1% CBD groups, 62.5 mg of 5% IMQ cream was applied first; then, 4 h later, 62.5 mg of white petrolatum was administered to the IMQ group, 120 mg of 0.05% clobetasol was administered to the Clobetasol group, and 0.01% and 0.1% CBD creams were administered to the 0.01% and 0.1% CBD groups, respectively (Figure 1).

The Psoriasis Area and Severity Index (PASI)—which assesses skin erythema, scaling, and thickness—was used to evaluate and rank the intensity of the psoriasis-like lesions on Day 6. The PASI score is calculated by evaluating skin color (erythema), skin scaling, and changes in skin thickness from a baseline measurement taken before imiquimod administration (healthy skin) [21]. The assessment of skin color (erythema), scaling, and thickness was performed visually by three dermatologists, as part of the scoring process. Each of the three readouts are scored from 0 (no incidence) to 3 (most severe) points, with the final score ranging from 0 to 9 points. The assessment of skin color (erythema), scaling, and thickness was performed visually by three dermatologists.

### 2.3. Quantitative Real-Time PCR in Mouse Dorsal Skin Tissue

After the mice were euthanized, tissue samples were obtained from the dorsal skin using a 5 mm biopsy punch. The obtained tissues were then homogenized in TRIzol (Invitrogen, Carlsbad, CA, USA) to facilitate the extraction of RNA. After homogenization, chloroform was added to the homogenate, and this was followed by thorough mixing. The mixture was incubated at room temperature for 15 min. Subsequently, the samples were centrifuged at 13,000 rpm for 15 min at 4 °C. After centrifugation, the upper aqueous phase containing the RNA was carefully separated and collected for further purification and analysis. The quality of RNA samples was assessed using NanoDrop (Thermo Fisher Scientific, Waltham, MA, USA). After RNA quantification, the synthesized primers were diluted and mixed with Power SYBR^®^ Green PCR Master Mix (Takara Biomedical Inc., Shiga, Japan) before quantitative real-time PCR analysis was performed using a CFX-96 thermocycler (Bio-Rad Laboratories, Hercules, CA, USA). The PCR conditions to amplify all genes were as follows: 10 min at 95 °C, followed by 45 cycles of 15 s at 95 °C and 30 s at 60 °C. Expression data were calculated from cycle threshold (Ct) values using the ΔCt quantification method [22]. For real-time PCR, relative mRNA values were normalized to the control value of murine β-actin. The oligonucleotide sequences used were the primer sequences seen in Table 1.

### 2.4. Histological Analysis

Skin tissue samples were fixed in 4% formaldehyde, embedded with paraffin wax, and sectioned into 4 μm slices. Sections were stained by hematoxylin and eosin (H and E). Epidermal thickness was measured at 15 spots on the epidermis in each slide.

### 2.5. Immunohistochemical Analysis

For immunohistochemical analysis, formaldehyde-fixed slides were deparaffinized and rehydrated, antigen retrieval was performed using heated citrate buffer pH6 (Agilent Technologies, Inc., Santa Clara, CA, USA), and the slides were incubated in peroxidase-blocking solution (Agilent Technologies, Inc., Santa Clara, CA, USA). The sections were incubated with primary antibodies (Table 2) overnight at 4 °C in a humid chamber. Horseradish peroxidase (HRP)-conjugated secondary antibody was detected using the Dako REAL™ EnVision/HRP kit (Agilent Technologies, Inc., Santa Clara, CA, USA) at room temperature. Sections were visualized with substrate–chromogen solution and then counterstained with Mayer’s hematoxylin (Agilent Technologies, Inc., Santa Clara, CA, USA).

### 2.6. Western Blotting in Mouse Dorsal Skin Tissue

Protein was extracted from mouse dorsal skin tissue, and the protein concentration was measured using a BCA Protein Assay Kit II (Thermo Fisher Scientific, Waltham, MA, USA). Proteins (20 μg) were separated via electrophoresis and transferred onto polyvinylidene fluoride membranes (MilliporeSigma, St. Louis, MO, USA). Membranes were blocked with 5% skim milk or 5% BSA in PBST. The primary antibodies (Table 2) were incubated overnight at 4 °C. The membranes were then analyzed using the appropriate secondary antibodies (Table 2). The visualization of the observed bands was quantified using ECL substrate (Thermo Fisher Scientific, Waltham, MA, USA) in conjunction with the Amersham™ Imager 600 (GE Healthcare, Chicago, IL, USA). The intensity of all observed bands was quantified using the ImageJ Fiji software (Version 1.54f; WS Rasband, U.S. National Institutes of Health, Bethesda, MD, USA) (http://imagej.net/Fiji/Downloads [accessed on 18 October 2023]).

### 2.7. Digital Analysis of Immunohistochemistry (IHC) Images

IHC-stained tissue samples were digitized using an optical microscope (DM2500 LED; Leica Microsystems, Wetzlar, Germany). The levels of STAT3, P-STAT3, and JAK2 expression, respectively, were quantified using a semi-quantitative protein-expression measurement approach, as described in the reference provided [23]. The ImageJ Fiji software program was used.

### 2.8. Statistical Analysis

Statistical analysis involved the use of one-way analysis of variance, followed by Tukey’s multiple-comparisons test. Unpaired *t* tests were employed for group comparisons. Graphs were generated using GraphPad Prism 5 software (GraphPad Software, La Jolla, CA, USA). All data are presented as mean ± standard error of the mean (SEM) values. Statistical significance was determined at *p* < 0.05 (* *p* < 0.05, ** *p* < 0.01, and *** *p* < 0.001 compared to the Control group or the IMQ group).

## 3. Results

### 3.1. CBD Alleviated Psoriasis Severity in the IMQ-Induced Psoriasis Mouse Model

**Figure 2:**
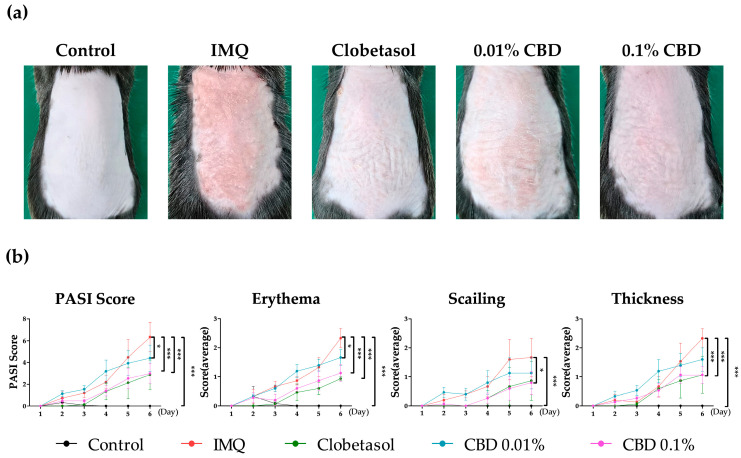
The impact of CBD cream on skin lesions in a psoriasis mouse model. (**a**) An image of the dorsal skin of a mouse on Day 6 is shown. (**b**) The PASI score, encompassing erythema, scaling, and thickness, is shown for Days 1–6. Data are presented as mean ± SEM values. * *p* < 0.05 and *** *p* < 0.001 compared to the Control group or IMQ group. CBD, cannabidiol; PASI, Psoriasis Area and Severity Index; IMQ, imiquimod; SEM, standard error of the mean.

To explore CBD’s impact on psoriasis pathophysiology, we induced psoriasis in mice using IMQ and subsequently applied CBD at concentrations of 0.01% and 0.1% to the affected dorsal skin. In photographic observations, distinct erythema and keratinization were evident in the IMQ group compared to the Control group. Conversely, the CBD-treated groups exhibited milder skin inflammation and improved phenotypic changes relative to the IMQ group (Figure 2a).

In the IMQ group, the PASI, erythema, scaling, and thickness scores were 6.33 (±2.31), 2.33 (±0.58), 1.67 (±1.15), and 2.33 (±0.58) points, respectively. In contrast, the 0.01% CBD group showed lower scores of 4.40 (±2.09) points for the PASI score, 1.67 (±0.46) points for erythema, 1.13 (±0.95) points for scaling, and 1.60 (±0.69) points for thickness. The 0.1% CBD group demonstrated further reductions, with scores of 3.00 (±1.56) points for the PASI, 1.13 (±0.50) points for erythema, 0.80 (±0.69) points for scaling, and 1.07 (±0.50) points for thickness. Significant reductions were observed in the PASI and erythema scores in the 0.01% CBD group (*p* < 0.05), with more pronounced decreases noted in the 0.1% CBD group (*p* < 0.001) compared to the IMQ group. The scaling score showed a significant decrease only in the 0.1% CBD group (*p* < 0.05), while the thickness score demonstrated a significant reduction in both the 0.01% CBD group (*p* < 0.05) and the 0.1% CBD group (*p* < 0.001) (Figure 2b).

**Figure 3:**
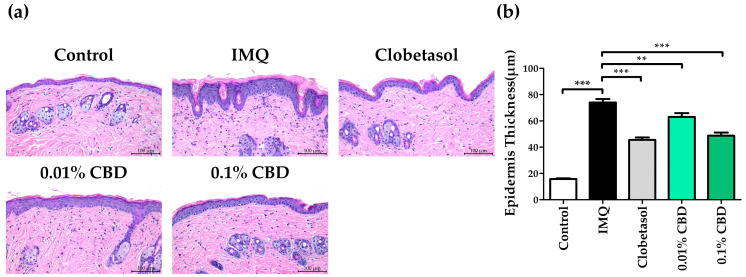
Comparison of epidermal thickness and H and E staining in a psoriasis mouse model treated with CBD. (**a**) Representative images of H and E staining of mouse dorsal skin. (**b**) Epidermal thickness change in each group. Data are presented as mean ± SEM values. ** *p* < 0.01 and *** *p* < 0.001 compared to the Control group or IMQ group. CBD, cannabidiol; H and E, hematoxylin and eosin; IMQ, imiquimod; SEM, standard error of the mean.

To further confirm the therapeutic effect of CBD, we performed H and E staining to assess changes in skin thickness in a psoriasis mouse model. The average epidermal thickness in the Control group was measured at 15.83 (±2.71) μm, while in the IMQ group, it was significantly greater at 74.02 (±17.84) μm, representing an approximately five-fold increase (*p* < 0.001). Comparatively, the 0.01% CBD group exhibited a notable reduction, to 63.00 (±19.88) μm (*p* < 0.01), compared to the IMQ group. Meanwhile, the 0.1% CBD group showed a significant decrease to 48.80 (±15.53) μm (*p* < 0.001) relative to the IMQ group (Figure 3a,b).

### 3.2. CBD Inhibited Inflammatory Cytokine Expression in a Psoriasis Mouse Model

**Figure 4:**
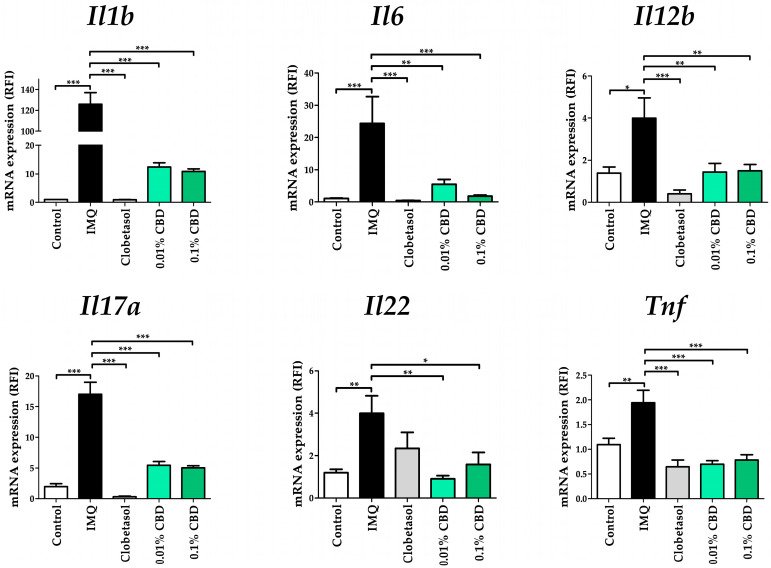
Expression of mRNA levels resulting from CBD treatment in a psoriasis mouse model. Effects of CBD on *Il1b*, *Il6*, *Il12b, Il17a*, *Il22*, and *Tnf* mRNA expression in a psoriasis mouse model. Total RNA was extracted and analyzed via quantitative real-time PCR. Each gene’s expression was normalized to *Actb* expression. Data are presented as mean ± SEM values from five mice. Statistical significance was indicated as * *p* < 0.05, ** *p* < 0.01, and *** *p* < 0.001 compared to the Control group or the IMQ group. CBD, cannabidiol; IMQ, imiquimod; SEM, standard error of the mean.

To elucidate CBD’s mechanism of action, we examined its effects on psoriasis-related cytokine mRNA levels. To this end, we analyzed the expression of specific cytokines, including *Actb*, *Il1b*, *Il6*, *Il12b*, *Il17a*, *Il22*, and *Tnf*. In the IMQ group, *Il1b* (*p* < 0.001), *Il6* (*p* < 0.001), and *Il17a* (*p* < 0.001) expression levels showed a greater than 15-fold increase compared to those in the Control group. Furthermore, *Il12b* (*p* < 0.05) and *Il22* (*p* < 0.01) expressions were upregulated approximately four-fold, while *Tnf* (*p* < 0.01) expression was upregulated approximately two-fold. In the 0.01% CBD group, the expression of *Il1b* was decreased approximately 10-fold (*p* < 0.001), that of *Il6* was decreased five-fold (*p* < 0.01), that of *Il12b* was decreased two-fold (*p* < 0.01), that of *Il17a* was decreased three-fold (*p* < 0.001), that of *Il22* was decreased four-fold (*p* < 0.01), and that of *Tnf* was decreased two-fold (*p* < 0.001) compared to those in the IMQ group, respectively. In the 0.1% CBD group, *Il1b* and *Il6* expression levels were decreased approximately 12-fold (*p* < 0.001), while that of *Il12b* was decreased 12-fold (*p* < 0.01), that of *Il17a* was decreased three-fold (*p* < 0.001), that of *Il22* was decreased two-fold (*p* < 0.05), and that of *Tnf* was decreased two-fold (*p* < 0.001) compared to those in the IMQ group (Figure 4).

### 3.3. CBD Alleviates Psoriasis by STAT3–JAK2 Pathway Blockage in a Psoriasis Mouse Model

**Figure 5:**
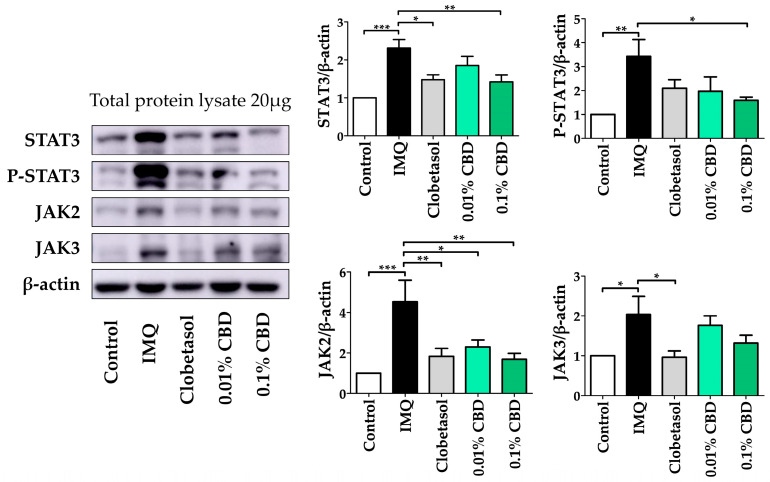
Treatment with CBD reduces the JAK–STAT protein concentration in a mouse model of psoriasis. STAT3, P-SATAT3, JAK2, and JAK3 protein expression after CBD treatment in a psoriasis mouse model. Immunoblotting intensity was calculated using ImageJ Fiji software (version 1.54f). Data are presented as mean ± SEM values. * *p* < 0.05, ** *p* < 0.01, and *** *p* < 0.001 compared to the Control group or the IMQ group. CBD, cannabidiol; IMQ, imiquimod; JAK, Janus kinase; SEM, standard error of the mean; STAT, signal transducer and activator of transcription.

We investigated the inhibitory effect of CBD on JAK–STAT signaling pathway activation in a psoriasis mouse model. Compared to the Control group, the IMQ-induced group showed a significant increase in the expression levels of STAT3 (*p* < 0.001), P-STAT3 (*p* < 0.01), JAK2 (*p* < 0.001), and JAK3 (*p* < 0.05) by more than two-fold each, respectively. In contrast, the 0.01% CBD group exhibited a significant decrease in JAK2 expression by approximately two-fold (*p* < 0.05). Moreover, the 0.1% CBD group demonstrated significant reductions in STAT3 (*p* < 0.01), P-STAT3 (*p* < 0.05), and JAK2 (*p* < 0.01) expression. Although not statistically significant, there was a tendency for STAT3 and JAK3 levels to decrease in the CBD-treated group (Figure 5). In contrast, TYK2 was not inhibited by CBD.

**Figure 6:**
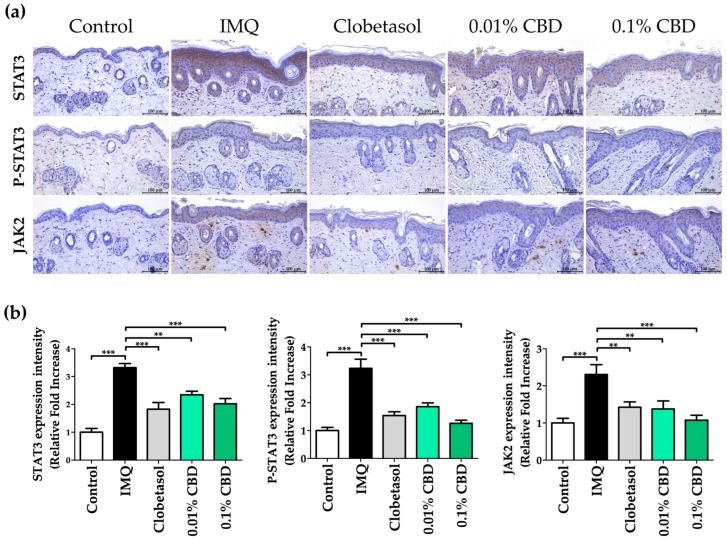
IHC staining analysis of skin lesions in a psoriasis mouse model treated with CBD. (**a**) IHC staining and (**b**) quantification of the JAK–STAT pathway protein expression in a psoriasis mouse model. Original magnification, ×200; scale bar = 100 μm. Data are presented as mean ± SEM values. ** *p* < 0.01 and *** *p* < 0.001 compared to the Control group or IMQ group. CBD, cannabidiol; IHC, immunohistochemistry; IMQ, imiquimod; JAK, Janus kinase; SEM, standard error of the mean; STAT, signal transducer and activator of transcription.

Following IHC staining, we detected STAT3 in the cytoplasmic region of the epidermis, P-STAT3 in the nuclear region of the epidermis, and JAK2 in both the cytoplasmic region of the epidermis and the nuclear region of the dermis, respectively (Figure 6a). The IMQ group showed a greater than two-fold increase in the expression levels of STAT3 (*p* < 0.001), P-STAT3 (*p* < 0.001), and JAK2 (*p* < 0.001) compared to those in the Control group, with all increases being statistically significant. Meanwhile, the 0.01% CBD group exhibited a significant reduction in the expression levels of STAT3 (*p* < 0.01), P-STAT3 (*p* < 0.001), and JAK2 (*p* < 0.01), and the 0.1% CBD group demonstrated a significant decrease in the expression levels of STAT3 (*p* < 0.001), P-STAT3 (*p* < 0.001), and JAK2, respectively (*p* < 0.001) (Figure 6b).

## 4. Discussion

This study shows that CBD significantly inhibits the JAK2 signaling pathway without any apparent effect on TYK2 or JAK3 expression. JAK2 is pivotal in the signaling pathways of numerous cytokines crucial for inflammatory and immune responses; specifically, JAK2 is closely involved in the activation of the IL-23/IL-17 axis, a key pathway implicated in the pathogenesis of psoriasis [24]. Psoriasis involves an aberrant immune response characterized by keratinocyte hyperproliferation and infiltration of inflammatory cells into the skin. Central to this process is the IL-23/IL-17 axis, which promotes the differentiation and proliferation of Th17 cells, leading to the production of inflammatory cytokines such as IL-17, IL-22, and IL-1β. Activation of JAK2 occurs following cytokine binding to its receptor, triggering STAT3 phosphorylation. The phosphorylation of STAT3 promotes dimerization and translocation to the nucleus, resulting in the expression of genes involved in inflammatory responses [25]. By inhibiting JAK2, CBD effectively reduces the phosphorylation of STAT3, thereby decreasing the expression of inflammatory cytokines. This contributes to the alleviation of psoriasis symptoms by suppressing keratinocyte proliferation and inflammation.

Several JAK2 inhibitors have been developed and are currently used in clinical practice for the treatment of psoriasis [26]. JAK1/2 inhibitors such as ruxolitinib and baricitinib have shown effectiveness in reducing psoriasis symptoms by modulating the immune response [27,28,29]. The specificity of CBD in inhibiting JAK2, as observed in our study, suggests its potential as a targeted treatment for psoriasis and offers an alternative to currently available JAK inhibitors with broader activity profiles.

**Figure 7:**
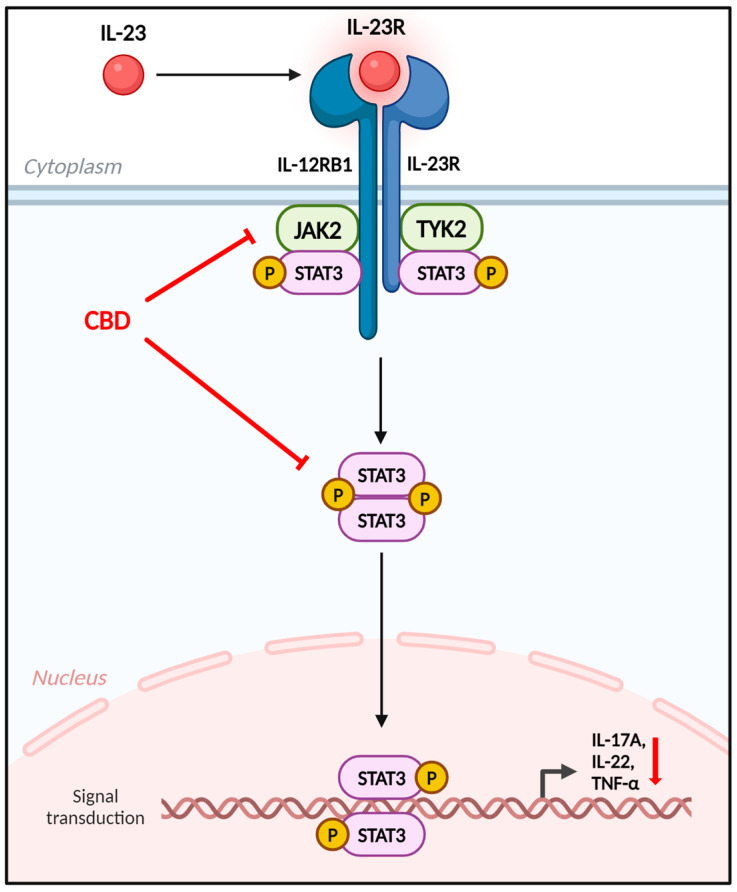
The role of CBD in modulating the psoriasis pathway. The IL-23 receptor, pivotal in the psoriasis pathway, promotes STAT3 phosphorylation and activation via JAK2 and TYK2. CBD inhibits JAK2 and STAT3, thereby suppressing key psoriasis-related cytokines, including IL-17A, IL-22, and TNF-α. CBD, cannabidiol.

In this study, we evaluated CBD’s efficacy in an IMQ-induced psoriasis mouse model, characterized by erythema, scaling, and skin thickening. Experimental groups included a Control group, an IMQ group, a Clobetasol group, and CBD therapy groups treated at 0.01% and 0.1% concentrations. CBD treatment, especially at 0.1%, significantly reduced psoriasis severity, as shown by a lower PASI score and reduced epidermal thickness. Molecular analyses revealed that CBD inhibited JAK2 expression, leading to reduced STAT3 and P-STAT3 protein levels. This inhibition resulted in a significant decrease in key inflammatory cytokines (*Il1b*, *Il6*, *Il12b*, *Il17a*, *Il22*, and *Tnf*) that are critical in psoriasis’ pathogenesis (Figure 7). These findings suggest that CBD effectively alleviates psoriasis symptoms by modulating the JAK2–STAT3 pathway and reducing activities of inflammatory cytokines, indicating its potential as a promising therapeutic agent for psoriasis.

JAK2 inhibitors are not only effective in treating psoriasis but are also used in several other skin diseases characterized by dysregulated immune responses. For example, the effectiveness of JAK2 inhibitors has been confirmed in chronic inflammatory skin diseases, such as atopic dermatitis, vitiligo, and alopecia [30,31,32,33]. Given CBD’s ability to inhibit JAK2, it is reasonable to believe that it could also be effective in treating these diseases. The anti-inflammatory properties of CBD, combined with its specific inhibition of JAK2, warrant further investigation into the use of CBD for treating atopic dermatitis and other immune-mediated conditions, such as alopecia. By targeting the underlying cytokine pathways involved in these diseases, JAK2 inhibition by CBD could potentially reduce inflammation, providing a novel therapeutic approach for these conditions. Our study lays the groundwork for exploring the broader dermatological applications of CBD, particularly in conditions where JAK2 plays a critical role in disease pathogenesis. CBD’s potential as a JAK2 inhibitor opens new avenues for the development of targeted therapies for a variety of inflammatory skin diseases, offering a safer and more specific alternative to existing treatments.

The development of new topical formulations for psoriasis has been relatively stagnant, with most efforts focusing on corticosteroids and vitamin D analogs [34]. While these agents are effective in reducing inflammation and the hyperproliferation of keratinocytes, they come with significant side effects and limitations. Topical corticosteroids, for instance, can cause skin atrophy, telangiectasia, and systemic effects with long-term use, and their effectiveness in managing psoriasis is further reduced by the potential for tachyphylaxis, in which the drug’s effectiveness diminishes with continued use [35,36].

Vitamin D analogs, such as calcipotriol, are often used as a steroid-sparing option in the treatment of psoriasis. However, excessive use can lead to irritation and hypercalcemia [37,38]. The lack of new, effective topical agents with better safety profiles underscores the need for innovative treatments that can provide lasting relief without the associated risks. As demonstrated in our study, CBD emerges as a promising candidate for topical psoriasis treatment. Its ability to inhibit JAK2 and modulate inflammatory pathways without serious side effects offers significant advantages over current treatments. Previous studies have highlighted the safety of CBD, noting minimal side effects [39]. The anti-inflammatory and immunomodulatory qualities of CBD position it favorably for creating novel topical treatments that can overcome the shortcomings of current therapies.

Although our study had promising results, there are some limitations to consider. The dependent use of an IMQ-induced psoriasis mouse model, the short-term treatment duration, and potential side effects require further investigation. Future studies should focus on long-term use, different models, and investigation of side effects to fully elucidate the therapeutic potential and safety of CBD in the treatment of psoriasis. Overall, CBD shows great promise, but continued research is important to ensure effective and safe application in the treatment of psoriasis and other inflammatory skin diseases.

## 5. Conclusions

Our results showed that CBD treatment markedly alleviated psoriasis symptoms in an IMQ-induced mouse model by targeting the JAK2–STAT3 signaling pathway. Therefore, CBD may have beneficial effects in treating psoriasis.

## Figures and Tables

**Table 1:** List of mouse primer sequences.

Gene	Forward	Reverse
*Il1b*	TGCCACCTTTTGACAGTGAT	AGTGATACTGCCTGCCTGAA
*Il6*	CCCCAATTTCCAATGCTCTCC	AGGCATAACGCACTAGGTTT
*Il12* *b*	CAGGCTGGACTGCATGATAG	CCAAGAAGGTAAGCAACCGA
*Il17* *a*	ATCCCTCTGTGATCTGGGAA	GCATCTTCTCGACCCTGAAA
*Il22*	TTCCGAGGAGTCAGTGCTAA	GAGTTTGGTCAGGAAAGGCA
*Tnf*	ATGTCCATTCCTGAGTTCTG	AATCTGGAAAGGTCTGAAGG
*Actb*	TGTGATGGTGGGAATGGGTCAGAA	TGTGGTGCCAGATCTTCTCCATGT

**Table 2:** Primary antibodies used in immunohistochemistry staining and western blotting.

Assay	Antibody	Dilution	Cat No.	Source
WB	β-actin	1:2500	#3700	Cell Signaling Technology^®^
WB/IHC	STAT3	1:1000/1:300	#9139	Cell Signaling Technology^®^
WB/IHC	P-STAT3	1:1000/1:200	#9145	Cell Signaling Technology^®^
WB/IHC	JAK2	1:1000	#3230	Cell Signaling Technology^®^
WB	JAK3	1:1000	#8863	Cell Signaling Technology^®^
WB	Goat Anti-Mouse IgG antibody (HRP)	1:4000	GRX213111-01	GeneTex
WB	Goat Anti-Rabbit IgG antibody (HRP)	1:4000	GRX213110-01	GeneTex

HRP, horseradish peroxidase; IgG, immunoglobulin G; IHC, immunohistochemistry; WB, western blotting.

## Data Availability

The presented data in this study can be found within the article.
